# Frequent Attendance to the Emergency Department after Release from Prison: a Prospective Data Linkage Study

**DOI:** 10.1007/s11414-019-09685-1

**Published:** 2019-12-09

**Authors:** Amanda Butler, Alexander D. Love, Jesse T. Young, Stuart A. Kinner

**Affiliations:** 1 Faculty of Health Sciences, Simon Fraser University, 313-1286 14th Avenue West, Vancouver, BC V6H 1P9 Canada; 2 Melbourne School of Population and Global Health, The University of Melbourne, Melbourne, VIC Australia; 3 Centre for Adolescent Health, Murdoch Children’s Research Institute, Melbourne, VIC Australia

**Keywords:** Emergency department, Mental illness, Dual diagnosis, Prisons, Re-entry

## Abstract

The aim of this paper was to identify characteristics and predictors of frequent emergency department (ED) use among people released from prisons in Queensland, Australia. Baseline interview data from a sample of sentenced adults were linked to ED and hospital records. The association between baseline characteristics and frequent ED attendance was modelled by fitting multivariate logistic regression models. Participants who had ≥ 4 visits to the ED in any 365-day period of community follow-up were defined as frequent attenders (FA). The analyses included 1307 people and mean follow-up time in the community was 1063 days. After adjusting for covariates, those with a dual diagnoses of mental illness and substance use (RR = 2.42, 95% CI 1.47–3.99) and those with mental illness alone (RR = 2.47, 95% CI 1.29–4.73) were at higher risk of frequent ED attendance, compared with those with no disorder. Future research should assess whether individually tailored transition supports from prison to community reduce the frequency of ED use among this population.

## Introduction

Emergency department (ED) overcrowding has become a widespread problem in Australia and other high-income countries such as Canada^1^ and the USA.^2, 3^ EDs in Australia are funded through state and territory health authorities, and are mostly embedded within public hospitals.^4^ Because EDs are a high-cost healthcare setting, efforts have been concentrated on reducing avoidable utilisation and streamlining patient flow^5^; examples of which include cost sharing, strengthening primary care and care coordination, diversion efforts (such as telephone triage), case-management, rapid access teams, waiting room nurses, and education and self-management support.^6^ While many of these initiatives have proven successful at reducing ED presentations and time delays,^7^ little research has focused on the characteristics and needs of the highest utilisers of the ED.

Frequent users of the ED, commonly referred to as *frequent attenders* (FA), have come under increased scrutiny, primarily because of the disproportionate burden they place on ED resources.^8–10^ A systematic review of 25 US studies estimated that FA make up 4.5–8.0% of the overall ED population, and account for 21–28% of all ED visits.^8^ Operational definitions of FA vary considerably, ranging from 3 to 10 visits in a 12-month period, often without a clear rationale for this cut-off.^10, 11^ The arbitrariness of the threshold for FA has been widely acknowledged, yet to our knowledge, only one study has attempted to define FA empirically. A study of 75,141 ED users in the UK found that ‘chance’ attenders (where events causing the patient to attend are independent, random events) would be expected to present on fewer than four occasions per year,^11^ suggesting that the minimum threshold for FA should be four or more ED events per year. However, no study has demonstrated an objective threshold where differences in demographic and health-related characteristics differ markedly by frequency of ED use. In other words, as ED visits increase, so does the proportion of individuals with poor mental and physical health.^10^ The considerable variability in operationalised definitions of FA has made it difficult to compare or integrate results from various studies.

Assumptions about patients who use the ED frequently have been largely based on anecdote rather than evidence.^8^ As such, FA remain a contentious issue among practitioners and policy-makers; some have speculated that frequent visits are avoidable and largely reflect non-emergency care needs,^12^ while others have suggested that ED diversion is not preferred (and in many cases, not feasible) for marginalised populations.^13, 14^ Furthermore, there is a lack of clarity about what constitutes ‘avoidable’ ED attendance, and little data around the effectiveness of primary care interventions to reduce ED attendance. Studies conducted across several countries and health systems have found similar FA patient characteristics including psychosocial vulnerability, poverty and homelessness, mental illness, substance use, chronic physical conditions, and premature mortality.^15–17^

We are aware of only a handful of studies internationally (and none in Australia) that have looked at frequent ED attendance among people who have experienced incarceration, a particularly vulnerable subpopulation. The period immediately following release from prison is associated with increased risk of preventable morbidity and mortality, injury including suicide, self-harm, and drug overdose, decompensation of disease, and hospitalisation.^18–25^ Being able to identify people who are at high risk for frequent ED attendance would allow justice and healthcare professionals to proactively provide targeted support.

In a large, representative cohort of adults released from prisons in Queensland, Australia, this study aimed to (1) quantify and describe instances of frequent ED attendance after release from prison; (2) determine clinical and social characteristics of frequent ED attenders; and (3) identify predictors of frequent ED attendance.

## Methods

### Participants

This study involved secondary analysis of data originally collected as part of a randomised trial. Baseline survey data were linked, retrospectively and prospectively, with administrative health and correctional records. Participants in this study were 1325 incarcerated adults (≥ 18) who were recruited into the *Passports* study: a randomised controlled trial of a low-intensity intervention designed to increase healthcare utilisation following release from prison.^26^ Participants were recruited within 6 weeks of expected release from their index incarceration (i.e. the incarceration during which they were recruited to the study) between 1 August 2008 and 31 July 2010. Except for intentional over-sampling of women, the cohort was representative of all people released from prisons in Queensland during the study period. Written, informed consent was obtained from all participants.

### Prison Medical Records

Prison medical records were coded by two trained graduate researchers using the International Classification of Primary Care, Second Edition (ICPC-2),^27^ which codes for problems and diagnoses managed, dates of contact, and the type of health professional seen.

### Baseline Characteristics

The baseline survey included the following measures: sex, age, Indigenous status, relationship status (stable/unstable), years of school completed (< 10, ≥ 10 years), pre-incarceration accommodation (stable/unstable), pre-incarceration employment status (employed/unemployed), and history of juvenile detention (yes/no).

Validated screening tools administered at baseline included the Kessler Psychological Distress Scale^28^ (K10—for predicting non-specific psychological distress), Patient Activation Measure^29^ (PAM—a measure of the confidence a patient has in managing their own health), and Enriched Social Support Inventory^30^ (ESSI—a measure of a patient’s perceived social support). Binary variables for the PAM and ESSI scores (below or above cohort median), and for the K10 distress categories (low or moderate; high or very high) were created.^31^ Valid tools for assessing substance use risk: the Alcohol, Smoking and Substance Involvement Screening Test^32^ (ASSIST—for ascertaining harmful substance use) and the Alcohol Use Disorder Identification Test^33^ (AUDIT-C—to assess alcohol consumption and alcohol-related problems) were included. Risk categories were combined to create binary variables for the ASSIST (low, moderate, or high) and the AUDIT-C scores (abstinent or low, moderate, high, or very high). Finally, the study included a variable for use of psychotropic medication (yes/no) using prison medical records.

### Data Linkage

Baseline data were probabilistically linked with state-wide ED, hospital, and correctional records, and national death records. Linkage was enhanced by the inclusion of all known aliases for participants, obtained from correctional records; this process has been shown to improve sensitivity without adversely affecting specificity.^34^ All ED presentations in the cohort in Queensland were identified and linked to baseline survey data. Variables obtained for each ED presentation included the International Classification of Diseases 10th edition (ICD-10-AM) diagnosis code^35^ assigned to the principal working diagnosis for each presentation, the date and time of each ED arrival and departure, the triage category (using the Australasian Triage Scale, ATS),^36^ departure destination, and departure status. Variables obtained from hospital records included primary and secondary diagnoses and external cause of morbidity ICD-10-AM codes, dates of admission and separation, and number of hospital bed days. From deterministic linkage with correctional records, our team obtained data on prior adult prison sentences, length of index prison sentence, and dates of reincarceration during follow-up. ED and hospital records were linked from 1 June 2002 to 31 July 2012 and 1 July 1999 to 31 July 2012, respectively. Correctional records included all prison admissions and releases from 1 September 2008 to 31 December 2013. Death records were linked from the date of index prison release to 31 May 2013. Follow-up for all participants was censored at 31 July 2012 (end of the ED and hospital record).

### Defining the Outcome: Frequent Attender

Time at risk was defined as days spent in the community after release from the index prison sentence, such that all periods of reincarceration (and ED visits during reincarceration) were excluded from follow-up. For participants who experienced subsequent releases from custody during the follow-up period, the follow-up period was truncated such that each participant’s follow-up consisted of the number of *community* days—that could include days occurring both before and after a reincarceration event. Deaths that occurred during follow-up were censored using death records. A 3-level variable was created which reflected frequency of ED attendance after release from prison: no attendance, low-frequency attenders (LFA), and frequent attenders (FA). Participants who had ≥ 4 visits to the ED in any 365-day period of community follow-up, and participants who had less than 365 days of community follow-up (*n* = 114) but had ≥ 4 visits to the ED during community follow-up, were defined as FA. This cut-off was chosen because it is empirically supported in the literature.^11^ The LFA group included those who did not meet the criteria of FA but who attended the ED at least once during their community follow-up period after release from their index prison sentence.

### Mental Disorder Status

We included pre-release mental disorder status which was constructed as a categorical variable with four levels: no disorder, mental illness only, substance use disorder only, and dual diagnosis. ICD-10-AM codes from ED presentations and hospital admissions and ICPC-2 codes from prison health service contacts for the period prior to and during the index prison sentence were used to ascertain these mutually exclusive categories. Further details of how this measure was derived are published elsewhere.^37^

### Statistical Analyses

Descriptive statistics were calculated for all measures. Differences between ED frequency categories on baseline characteristics and ED presentation characteristics (triage category, presenting problem, and discharge summary) during follow-up were compared using chi-square tests.

Univariate and multivariate multinomial logistic regression models were fit to estimate the association between baseline variables and ED frequency category. The multivariate model was adjusted for sex, age, Indigenous status, years of school completed, pre-incarceration employment status, pre-incarceration accommodation, relationship status at baseline, ASSIST scores (for methamphetamine, heroin and other opioids, and cannabis use), AUDIT-C score, ESSI score, PAM score, history of juvenile detention, K10 score, self-reported psychotropic medication use, any ED contact in the 12 months prior to index incarceration (ascertained using the ED record), mental disorder status (no disorder, mental disorder only, substance use disorder only, or dual diagnosis), index prison sentence length (≤ 90 days; 91–365 days; ≥ 366 days), incarceration during the follow-up period (any/none), and length of follow-up (in days).

All analyses were conducted using STATA version 14.2.^38^

### Ethical Considerations

The study was approved by the University of Queensland Behavioural and Social Sciences Ethical Review Committee (#2007000607), Queensland Health Human Research Ethics Committee (HREC/11/QHC/40), Queensland Corrective Services Research Committee, and Australian Institute of Health and Welfare Ethics Committee (EC2012/4/58).

## Results

After excluding individuals who were not released from prison during the study period, and those for whom linked ED data were unavailable (*n* = 18), our analyses included 1307 people (98.6%). The mean age at baseline was 32.7 years (interquartile range (IQR) 24–38 years), most of the cohort (*n* = 1030, 78.8%) was male, and 25.3% (*n* = 331) identified as Indigenous. Mean follow-up time after release from prison was 1063 days (range 7–1420).

The total number of ED visits in the follow-up period was 3484. The FA group (*n* = 236, 18.1% of the cohort) accounted for 64.7% of ED visits during the follow-up period. The LFA group (46.1% of the cohort) accounted for 35.3% of ED visits, and 35.8% of the cohort had no ED visits during follow-up. Characteristics of the cohort overall and by FA category are presented in Table 1.

**Table 1:** Cohort characteristics stratified by ED attender frequency

Baseline characteristic	No ED visits *n* = 468 (35.8%)	1–3 ED visits (LFA) *n* =603 (46.1%)	4+ ED visits (FA) *n* = 236 (18.1%)	Total cohort (*n* = 1307)	*p* value^a^
Average age at baseline (SD)	33.3 (11.59)	32.8 (11.23)	31.2 (9.10)	32.7 (11.03)	0.021^b^
Female	80 (17.1)	138 (22.9)	59 (25.0)	277 (21.2)	0.020
Indigenous	124 (26.5)	145 (24.1%)	62 (26.3)	331 (25.3)	0.615
Had unstable accommodation prior to incarceration	73 (15.6)	91 (15.1)	56 (24.1)	220 (16.9)	0.005
Stable relationship	262 (56.3)	347 (58.0)	148 (63.5)	757 (58.4)	0.187
Unemployed prior to incarceration	225 (48.1)	286 (47.5)	133 (56.4)	644 (49.3)	0.056
Completed < 10 years of school	179 (38.3)	285 (47.5)	99 (42.0)	563 (43.2)	0.010
High or very high psychological distress (K10)	112 (24.1)	148 (24.6)	79 (33.5)	339 (26.4)	0.016
PAM score below cohort median	218 (46.6)	258 (42.8)	107 (45.3)	583 (44.6)	0.450
ESSI score below cohort median	216 (46.2)	289 (47.9)	116 (49.2)	621 (47.5)	0.725
Has experienced juvenile detention	126 (26.9)	168 (27.9)	67 (28.4)	361 (27.6)	0.904
Attended the ED in the 12 months prior to incarceration ^c^	127 (27.7)	283 (48.1)	159 (67.4)	569 (44.4)	< 0.001
Index prison sentence					
0–90 days	101 (21.6)	185 (30.7)	83 (35.2)	369 (28.2)	< 0.001
91–365 days	259 (55.3)	290 (48.1)	121 (51.3)	670 (51.3)	
> 365 days	108 (23.1)	128 (21.2)	32 (13.6)	268 (20.5)	
Taking psychotropic medication at baseline	99 (21.2)	183 (27.9)	94 (39.8)	361 (27.6)	< 0.001
ASSIST risk of harmful substance use					
Alcohol					
Moderate or high risk	272 (58.1)	382 (63.4)	163 (69.1)	817 (62.5)	0.015
Methamphetamine					
Moderate or high risk	146 (31.2)	241 (40.0)	114 (48.3)	501 (38.3)	< 0.001
Heroin or other opioid					
Moderate or high risk	80 (17.1)	124 (20.6)	73 (30.9)	277 (21.2)	< 0.001
Cannabis					
Moderate or high risk	191 (40.8)	282 (46.8)	133 (56.4)	606 (46.4)	< 0.001
Mental health status					
Dual diagnosis	64 (13.7)	121 (20.1)	92 (39.0)	277 (21.2)	< 0.001
MI alone	36 (7.7)	41 (6.8)	22 (9.3)	99 (7.6)	
SUD alone	90 (19.2)	160 (26.5)	64 (27.1)	314 (24.0)	
No MI or SUD	278 (59.4)	281 (46.6)	58 (24.6)	617 (47.2)	

^a^Pearson chi-squared test; ^b^Unadjusted linear regression; ^c^ED data was available starting from June 2002. Twenty-four participants had index sentences prior to June 2002, so for this variable *n* = 1283

*ED*, emergency department; *LFA*, low frequency attender; *FA*, frequent attender; *SD*, standard deviation; *K10*, Kessler 10; *PAM*, patient activation measure; *ESSI*, ENRICHD Social Support Inventory; *ASSIST*, Alcohol, Smoking and Substance Use Involvement Screening Test; *MI*, mental illness; *SUD*, substance use disorder

Thirty-nine percent of the FA group had a dual diagnosis, compared with 20.1% of LFA and 13.7% of non-attenders (*p* < 0.001). Nearly one-quarter (24.1%) of the FA group were unstably housed prior to incarceration compared with 15.1% of LFA and 15.6% of non-attenders (*p* = 0.05). Approximately 40% of the FA group were taking psychotropic medication in prison, compared with 27.9% of LFA and 21.2% of non-attenders (*p* < 0.001).

Associations between baseline characteristics and frequency of ED attendance are summarised in Table 2. After adjusting for model covariates, those with dual diagnosis and those with mental illness alone were at higher risk of frequent ED attendance, compared with those with no mental disorder (RR = 2.42, 95% CI 1.47–3.99 and RR = 2.47, 95% CI 1.29–4.73, respectively). Risk of FA was also greater for those who had presented to the ED prior to incarceration (RR = 1.76, 95% CI 1.24–2.50).

**Table 2:** Unadjusted and adjusted relative risk of being a frequent attender

Characteristic at baseline	Model 1: Unadjusted^a^				Model 2: Adjusted^ab^			
	Non-attender		Frequent attender		Non-attender		Frequent attender	
	RRR (95% CI)	*p* value	RRR (95% CI)	*p* value	RRR (95% CI)	*p* value	RRR (95% CI)	*p* value
Age								
Under 25 years	0.87 (0.64–1.19)	0.392	1.12 (0.75–1.67)	0.585	1.05 (0.73–1.50)	0.796	1.01 (0.65–1.59)	0.951
Aged 25–34	0.81 (0.61–1.07)	0.140	1.41 (0.99–2.00)	0.057	0.91 (0.66–1.25)	0.551	1.17 (0.79–1.73)	0.431
Age ≥ 35 years	1	–	1	–	1	–	1	–
Sex								
Male	1	–	1	–	1	–	1	–
Female	0.69 (0.51–0.94(	0.020	1.12 (0.79–1.60)	0.516	0.74 (0.52–1.07)	0.114	0.85 (0.56–1.28)	0.433
Indigenous status								
Non-indigenous	1	–	1	–	1	–	1	–
Indigenous	1.14 (0.86–1.50)	0.360	1.13 (0.80–1.59)	0.502	1.40 (0.98–1.98)	0.068	1.10 (0.72–1.68)	0.668
Accommodation status								
Stable	1	–	1	–	1	–	1	–
Unstable	01.04 (0.744–1.45)	0.817	0.79 (1.23–2.60)	0.002	1.15 (0.79–1.67)	0.466	1.53 (1.01–2.31)	0.042
Relationship status								
Stable relationship	1	–	1	–	1	–	1	–
Single	0.93 (0.70–1.19)	0.582	1.26 (0.92–1.72)	0.148	0.98 (0.74–1.30)	0.882	1.16 (0.82–1.65)	0.404
Employment status								
Employed	1	–	1	–	1	–	1	–
Unemployed	1.02 (0.80–1.30)	0.854	1.43 (1.05–1.93)	0.022	1.25 (0.94–1.65)	0.127	1.21 (0.86–1.70)	0.264
Education completed								
≥ 10 years	1	–	1	–	1	–	1	–
< 10 years	0.69 (0.54–0.89)	0.003	0.80 (0.59–1.09)	0.148	0.67 (0.50–0.88)	0.004	0.70 (0.50–0.98)	0.040
K10 psychological distress score								
Low/moderate	1	–	1	–	1	–	1	–
High or very high	0.97 (0.73–1.29)	0.839	1.54 (.10–2.14)	0.010	1.13 (0.83–1.56)	0.426	1.23 (0.86–1.77)	0.261
PAM activation score								
≥ Cohort median score	1	–	1	–	1	–	1	–
< Cohort median for	1.17 (0.91–1.49)	0.215	1.11 (0.82–1.50)	0.503	1.38 (1.05–1.82)	0.020	0.94 (0.67–1.31)	0.697
ESSI score								
≥ Cohort median score	1	–	1	–	1	–	1	–
< Cohort median score	0.93 (0.73–1.19)	0.564	1.05 (0.78–1.42)	0.750	0.88 (0.66–1.17)	0.392	0.88 (0.62–1.25)	0.483
Had experience juvenile detention								
No	1	–	1	–	1	–	1	–
Yes	0.95 (0.73–1.25)	0.733	1.03 (0.73–1.43)	0.878	1.09 (0.79–1.50)	0.599	0.84 (0.58–1.23)	0.374
Attended the ED in the 12 months prior to incarceration								
No	1	–	1	–	1	–	1	–
Yes	0.41 (0.32–0.54)	< 0.001	2.33 (1.63–3.06)	< 0.001	0.42 (0.32–0.56)	< 0.001	1.76 (1.24–2.50)	0.002
Prison sentence length								
0–90 days	0.65 (0.45–0.92)	0.016	1.79 (1.12–2.86)	0.014	0.78 (0.52–1.18)	0.244	1.57 (0.94–2.61)	0.083
91–365 days	1.06 (0.78–1.43)	0.716	1.66 (1.13–2.86)	0.014	1.28 (0.90–1.81)	0.173	1.26 (0.79–2.02)	0.338
> 365 days	1	–	1	–	1	–	1	–
Taking psychotropic medication at baseline								
No	1	–	1	–	1	–	1	–
Yes	0.69 (0.522–0.923)	0.012	1.71 (1.25–2.35)	0.001	0.85 (0.60–1.20)	0.347	1.07 (0.73–1.58)	0.718
ASSIST risk of harmful substance use								
Alcohol								
Low risk	1	–	1	–	1	–	1	–
Moderate or high risk	0.80	0.082	1.29	0.119	0.86 (0.64–1.15)	0.305	1.18 (0.81–1.71)	0.387
Methamphetamine								
Low risk	1	–	1	–	1	–	1	–
Moderate or high risk	0.68 (0.53–0.88)	0.003	1.40 (1.04–1.90)	0.028	0.90 (0.65–1.23)	0.500	0.96 (0.66–1.39)	0.810
Heroin or other opioids								
Low risk	1	–	1	–	1	–	1	–
Moderate or high risk	0.80 (0.58–1.09)	0.152	1.73 (1.23–2.43)	0.002	1.26 (0.86–1.84)	0.239	1.33 (0.89–2.00)	0.159
Cannabis								
Low risk	1	–	1	–	1	–	1	–
Moderate or high risk	0.78 (0.61–1.00)	0.052	1.47 (1.09–2.00)	0.013	0.70 (0.70–1.26)	0.661	1.17 (0.82–1.67)	0.387
Mental health status								
No MI or SUD	1	–	1	–	1	–	1	–
Dual diagnosis	0.53 (0.38–0.76)	< 0.001	3.68 (2.49–5.45)	< 0.001	0.74 (0.49–1.14)	0.173	2.42 (1.47–3.99)	0.001
MI alone	0.89 (0.55–1.43)	0.642	2.60 (1.44–4.69)	0.002	1.05 (0.62–1.80)	0.849	2.47 (1.29–4.73)	0.007
SUD alone	0.57 (0.42–0.77)	< 0.001	1.94 (1.29–2.91)	< 0.001	0.72 (0.50–1.02)	0.064	1.39 (0.87–2.23)	0.167

^a^Low-frequency attender (1–3 ED visits) is the reference group. ^b^Additional adjustment variables included length of follow-up and reincarceration during the follow-up period

*RRR*, relative risk ratio; *K10*, Kessler 10; *PAM*, patient activation measure; *ESSI*, ENRICHD Social Support Inventory; *ASSIST*, Alcohol, Smoking and Substance Use Involvement Screening Test; *ED*, emergency department; *MI*, mental illness; *SUD*, substance use disorder

Characteristics of the ED events overall and by FA category are presented in Table 3. Approximately one-fifth (17.6%) of all ED visits among the FA group were for mental disorders (ICD-10-AM codes F01-F99, Chapter V), compared with 8.4% of visits among the LFA group. The FA group had more presentations for ‘factors influencing health and health status and health services’ (ICD-10-AM codes Z00-Z99, Chapter XXI) compared with the LFA group (22.5% vs. 16.9%, *p* < 0.001). The FA group had a higher proportion of category 5 triage codes (less urgent) compared with the LFA group (15.0% vs. 8.0%, *p* < 0.001). Most of the ED attendances did not result in a hospital admission (57.7% of the total visits resulted in a discharge) and there were no significant differences between the two groups in terms of documented discharge decisions.

**Table 3:** ED presentation characteristics stratified by ED attendance frequency category

ED presentation characteristic		ED visits among LFA *n* = 1228 (35.3%)	ED visits among FA *n* = 2250 (64.7%)	All ED visits *n* = 3484	*p* value
Triage category^a^	1	39 (3.2)	49 (2.2)	88 (2.5)	< 0.001
	2	146 (11.9)	323 (14.4)	469 (13.5)	
	3	465 (37.9)	806 (35.8)	1271 (36.5)	
	4	480 (39.1)	735 (32.7)	1215 (34.9)	
	5	98 (8.0)	337 (15.0)	435 (12.5)	
ICD-10 category	XIX: Injury, poisoning and certain other consequences of external causes	469 (38.1)	540 (24.0)	1009 (29.0)	< 0.001
	XXI: Factors influencing health and status and contact with health services^b^	208 (16.9)	507 (22.5)	715 (20.5)	< 0.001
	V: Mental and behavioural disorders	103 (8.4)	397 (17.6)	500 (14.4)	< 0.001
	XVIII: Symptoms, signs, and abnormal clinical and laboratory findings, not elsewhere classified	97 (7.9)	177 (7.9)	274 (7.9)	0.972
	XII: Diseases of the skin and subcutaneous tissue	76 (6.2)	132 (5.9)	208 (6.0)	0.701
Destination^c^	Left	185 (15.1)	387 (17.2)	572 (16.4)	0.265
	Discharged	720 (58.6)	1288 (57.2)	2008 (57.7)	
	Admitted	324 (26.4)	576 (25.6)	900 (25.9)	

^a^Triage data available for 3478 observations. Level 1: Immediately life-threatening; level 2: Imminently life-threatening; level 3: Potentially life-threatening; level 4: Potentially serious; level 5: Less urgent

^b^Chapter XXI includes a range of diagnostic codes for encounters other than illness and injury, including potential hazards related to communicable diseases, socioeconomic and psychological circumstances, and hazards related to family and personal history

^c^Documented discharge destination from ED records for 3480 events included here. Four observations were excluded because this information was missing, or the individual died in ED.

*ED*, emergency department; *LFA*, low frequency attender; *FA*, frequent attender

## Discussion

The aims of the study were to quantify and describe instances of frequent ED attendance after release from prison, determine the characteristics of frequent ED attenders, and identify predictors of frequent ED attendance. A minority of people in our cohort accounted for the majority of ED presentations after release from prison. Most ED visits were for Chapter XIX: ‘injury, poisoning and certain other consequences of external causes.’ However, the FA group were more likely than LFA to present for issues related to mental and behavioural disorders. Although there is little research on ED use among people released from prison, our findings are consistent with those from studies of community samples, where frequent ED attenders have significantly elevated rates of mental disorders compared with non-frequent attenders and non-users.^39–41^ The FA group were more likely than LFA to have their attendance triaged as category 5 on the ATS; this category includes chronic and minor conditions, medical certificates, contact for prescriptions refills only, social crisis, and well-known patients with chronic symptoms (collectively referred to as *clinico-administrative problems*).^36^ This is consistent with other studies which have found that FA have high rates of chronic disease such as heart disease and diabetes,^42, 43^ as well as psychosocial vulnerabilities such as homelessness and poverty.^15, 16^

A recent history of mental illness *alone* was predictive of FA, even after adjustment for covariates. Mental health services in many correctional settings are inadequate,^44, 45^ despite the high prevalence of mental illness among people in custody.^46–48^ In 2015, nearly half (49%) of prison entrants in Australia reported having been told by a health professional that they have a mental disorder (which could include a substance use disorder) and 27% reported currently taking psychotropic medication.^49^ Many people report that their mental health improves during a prison stay in Australia—prison offers a unique window of opportunity for assessment, intervention, and treatment for a highly socially marginalised population.^50^ Unfortunately, health gains made ‘behind bars’ are often lost after release from prison^51^ due, at least in part, to treatment interruption and inadequate transitional support. This is particularly true in Australia where the exclusion of people in prison from Medicare and the Pharmaceutical Benefits Scheme (PBS) poses a barrier to continuity of care.^49^

After adjusting for covariates, having a substance use disorder *alone* was not predictive of FA. One possible explanation for this is illicit substance use is a key target for criminogenic risk reduction programmes in correctional settings. Those with substance use disorder alone (as opposed to those which are ‘complicated’ by comorbid mental illness) may be more likely to succeed in achieving abstinence within traditional substance use treatment settings and be eligible for residential treatment programmes upon release from prison.^52, 53^ A 1-year prospective study of 565 males released from prison in the USA also found that self-reported drug abuse at baseline was not significantly related to ED utilisation.^54^

Finally, having a dual diagnosis was predictive of FA, even after adjustment for covariates. As a group, people with dual diagnosis are not well-managed through traditional community-based treatment, which puts them at higher risk for acute care contacts. A US study found that psychiatric patients with dual diagnosis had adjusted odds ratios ranging from 2.8–4.9 for several categorisations of frequent ED use.^40^ It is not uncommon for people with dual diagnosis to be excluded from alcohol and other drug services due to a lack of resources and expertise in treating co-occurring conditions.^52^ Furthermore, many people are released into the community on parole, which usually include conditions of abstinence from alcohol and other drugs (a violation of which could result in returning to custody). A US study of 8149 people released from prison on parole found that people with dual diagnosis were at elevated risk of having their parole revoked for a technical violation compared with those with no disorder, whereas those with a single disorder demonstrated no increased risk.^55^

### Strengths and Limitations

This is the first Australian study to examine patterns and characteristics of frequent ED use among people released from prison, an understudied population. The sample was broadly representative of all people released from prisons in Queensland during the study period.^26^ Rich baseline survey data, linked prospectively with state-wide emergency department records, permitted extensive adjustment for potential confounders. The composite measure for mental illness, substance use disorder, and dual diagnosis exposure was ascertained from a unique combination of retrospectively linked state-wide hospital, ED, and prison medical records. Our analyses were adjusted for episodes of reincarceration during follow-up, which are more common among frequent ED attenders,^56^ using prospectively linked correctional records. Our study also censored for deaths using linked prospective national death records.

The study has a few notable limitations. First, our follow-up period was relatively short—particularly since periods of reincarceration were removed for the purposes of defining FA (~ 9% of the sample did not have 365 community follow-up days). This prevented us from studying whether people who met our definition of FA immediately after release from prison remained a FA over time. Second, the findings may not necessarily be generalisable to other countries or jurisdictions, particularly those with different correctional and healthcare systems. In Australia, 94% of ED patients attend EDs embedded within public hospitals,^4^ and both patient characteristics and models of care coordination may differ in settings where a larger proportion of EDs are private. Our findings will require replication in other settings, ideally with longer follow-up periods. Thirdly, our definition of FA was not empirically derived, but was based on a recommended definition in the literature (≥ 4 events per year).^11^ Although people with dual diagnosis are at markedly increased risk of criminal justice involvement,^55, 57–59^ it will be important for future studies to compare patterns of ED utilisation among people with and without a history of criminal justice involvement.

## Implications for Behavioural Health

The unique challenges faced by individuals with dual diagnosis and criminal justice involvement cannot be overlooked—stigma, employment challenges, homelessness, difficulties adhering to treatment and parole conditions, social isolation and marginalisation, and medical comorbidity including chronic diseases.^60–62^ A fragmented and compartmentalised model of treatment continues to be implemented in many settings, such as Australia, Canada, and the USA, despite the growing evidence regarding the disproportionate adverse health, social, and criminal justice outcomes experienced by people with dual diagnosis.^37, 61–64^ Policy action and treatment for this group therefore needs to address the syndemic interaction of criminal behaviour, mental illness, and substance use disorders, as well as biopsychosocial and contextual vulnerabilities. A key challenge in implementing such policy responses is the need for multi-sectoral coordination across health, welfare, and criminal justice agencies. Further research, demonstrating the whole-of-government economic impacts of poor health outcomes for people with dual diagnosis after release from prison, may assist in making the case for multi-sectoral policy reform. Studies have demonstrated that healthcare interventions need to be available immediately upon release from prison for optimal impact, and that transitional care should be initiated *during* the incarceration period.^65^

FA are frequent users of not only the ED but also primary care and other acute health services,^15, 66^ suggesting that appropriateness of care—rather than simply access—may be a critical issue for this subgroup.^14^ Australian studies have shown that many FA are not suitable for diversion to general practice,^67^ such that reducing their ED use while ensuring that their needs are met in the ‘appropriate’ setting may be more difficult than simply triaging these patients and providing them with community-based resource information. Policy-makers should consider whether EDs could be better resourced to deliver care to vulnerable and complex patients, and proponents of diversion should focus on where people with complex needs ought to be diverted *to*.

The evidence-base concerning FA interventions is inconclusive, but adequate ED resourcing should be coupled with patient navigation support which accounts for individual-level needs.^42^ Multidisciplinary case management has received attention as a promising means to treat FA. In case management, a single point of contact (i.e. the case manager) is tasked with brokering access to care and guiding the FA through an individually tailored care plan, which may extend beyond the ED into the community.^68^ Research is required to determine best practices in client care for people released from prison, and in particular, those with mental illness and substance use issues.

Most people released from prison in Australia will have contact with an ED in the first year after release, and a subgroup of frequent attenders will account for the significant majority of ED visits among this population. Given the disproportionate burden on the healthcare system, and the elevated risk of adverse health outcomes for frequent ED users, efforts should be made to understand the characteristics and causes of frequent attendance to ED, and to accurately identify those at highest risk such that costly interventions can be targeted accordingly. People released from prison with mental illness and dual diagnosis are at increased risk of being frequent users of the ED. Future research should assess whether access to individually tailored transitional support, which combines public health and public safety practices, reduces the frequency of ED use among this population.
